# Novel Chemically Modified Curcumin (CMC) Derivatives Inhibit Tyrosinase Activity and Melanin Synthesis in B16F10 Mouse Melanoma Cells

**DOI:** 10.3390/biom11050674

**Published:** 2021-04-30

**Authors:** Shilpi Goenka, Francis Johnson, Sanford R. Simon

**Affiliations:** 1 Department of Biomedical Engineering, Stony Brook University, Stony Brook, NY 11794-5281, USA; sanford.simon@stonybrook.edu; 2 Department of Chemistry, Stony Brook University, Stony Brook, NY 11790-3400, USA; francis.johnson@stonybrook.edu; 3 Department of Pharmacological Sciences, Stony Brook University, Stony Brook, NY 11794-8651, USA; 4 Chem-Master International, Inc., Hauppauge, NY 11788, USA; 5 Department of Biochemistry and Cellular Biology, Stony Brook University, Stony Brook, NY 11794-5215, USA; 6 Department of Pathology, Stony Brook University, Stony Brook, NY 11794, USA

**Keywords:** curcumin, chemically modified curcumin, tyrosinase, enzyme kinetics, melanogenesis, α-glucosidase, cAMP, MITF

## Abstract

Skin hyperpigmentation disorders arise due to excessive production of the macromolecular pigment melanin catalyzed by the enzyme tyrosinase. Recently, the therapeutic use of curcumin for inhibiting tyrosinase activity and production of melanin have been recognized, but poor stability and solubility have limited its use, which has inspired synthesis of curcumin analogs. Here, we investigated four novel chemically modified curcumin (CMC) derivatives (CMC2.14, CMC2.5, CMC2.23 and CMC2.24) and compared them to the parent compound curcumin (PC) for inhibition of in vitro tyrosinase activity using two substrates for monophenolase and diphenolase activities of the enzyme and for diminution of cellular melanogenesis. Enzyme kinetics were analyzed using Lineweaver-Burk and Dixon plots and nonlinear curve-fitting to determine the mechanism for tyrosinase inhibition. Copper chelating activity, using pyrocatechol violet dye indicator assay, and antioxidant activity, using a DPPH radical scavenging assay, were also conducted. Next, the capacity of these derivatives to inhibit tyrosinase-catalyzed melanogenesis was studied in B16F10 mouse melanoma cells and the mechanisms of inhibition were elucidated. Inhibition mechanisms were studied by measuring intracellular tyrosinase activity, cell-free and intracellular α-glucosidase enzyme activity, and effects on MITF protein level and cAMP maturation factor. Our results showed that CMC2.24 showed the greatest efficacy as a tyrosinase inhibitor of all the CMCs and was better than PC as well as a popular tyrosinase inhibitor-kojic acid. Both CMC2.24 and CMC2.23 inhibited tyrosinase enzyme activity by a mixed mode of inhibition with a predominant competitive mode. In addition, CMC2.24 as well as CMC2.23 showed a comparable robust efficacy in inhibiting melanogenesis in cultured melanocytes. Furthermore, after removal of CMC2.24 or CMC2.23 from the medium, we could demonstrate a partial recovery of the suppressed intracellular tyrosinase activity in the melanocytes. Our results provide a proof-of-principle for the novel use of the CMCs that shows them to be far superior to the parent compound, curcumin, for skin depigmentation.

## 1. Introduction

Melanocytes originate from neural crest-derived melanoblasts and have a key role in the synthesis of melanin pigment within organelles called melanosomes, which are then secreted and transferred to keratinocytes [1]. Melanin is ubiquitously present in the skin, hair and eyes in mammals, where it provides UV photo-protection [2], free radical-scavenging activity and a host of other biological benefits [3]; however, the excessive production of melanin in the skin can lead to hyperpigmentation and is associated with skin disorders such as melasma, post-inflammatory hyperpigmentation (PIH) and lentigo senilis (LS), resulting in significant psychosocial burden. Mammalian tyrosinase (EC 1.14.18.1) is a copper-containing membrane-bound glycoprotein that is the key rate-limiting enzyme in the synthesis of the macromolecular pigment, melanin; it catalyzes the conversion of l-tyrosine to l-3,4-dihydroxyphenylalanine (l-DOPA) and subsequent conversion to Dopaquinone. Tyrosinase contributes to excessive production of dopaquinone from dopamine, resulting in neurotoxicity and leading to neurodegenerative changes that have been linked to Parkinson’s disease [4,5,6]. A closely related soluble tyrosinase in plants causes browning of fruits and vegetables during post-harvest processing, leading to poor shelf-life [7,8,9]. The enzyme also causes cuticle formation in insects [10]. Tyrosinase contains two copper atoms in its active site [11] and thus compounds which can chelate copper can inhibit tyrosinase activity. The most popular commercial tyrosinase inhibitors, such as kojic acid (KA), hydroquinone and arbutin (glycosylated hydroquinone) exhibit serious side-effects: KA causes pigmented contact dermatitis [12], hydroquinone is carcinogenic [13] and arbutin has potent genotoxicity [14]. These limitations have prompted a surge in identification of novel and natural plant-derived compounds that are associated with fewer adverse effects for reduction in food browning and for treatment of hyperpigmentation both in cosmetic and clinical settings.

Curcumin (diferuloylmethane; 1,7-bis(4-hydroxy-3-methoxyphenyl)-1,6-heptadien-3,5-dione) is a yellow-colored polyphenolic compound found in the spice turmeric (*Curcuma longa*); it has a rich history of use in the Asian and Indian subcontinents. Curcumin has been shown to possess multiple biological activities, among which are antioxidant, anti-inflammatory, anticancer and neuroprotective activities, to name a few; its uses have been reviewed extensively [15]. Turmeric extract comprises three major curcuminoids (curcumin I, curcumin II, and curcumin III), of which curcumin (curcumin I) is most abundant (77%), along with curcumin II (demethoxycurcumin, 17%) and curcumin III (bis-demethoxycurcumin, 3%) [16]. Commercial grade curcumin that has been used in many research studies is thus not chromatographically pure and usually consists of a mixture of curcumin I, II and III with various lesser amounts of other curcuminoids. Hence, true comparisons of structure-activity relationships (SAR) cannot be made from results using such mixtures. Additionally, the poor solubility and stability of curcumin present significant limitations for its use in formulations [17,18]. There have been some reports of curcumin analogs that display tyrosinase inhibiting activity [19,20].

Our group has reported on CMC2.24, a triketonic *N*-phenylaminocarbonyl derivative of bis-demethoxycurcumin, which exhibits enhanced stability and solubility compared to curcumin and has emerged as a ‘lead compound’ for several pharmacological applications such as treatment of anthrax by inhibition of the metalloprotease, lethal factor [21], treatment of cancers of the pancreas [22] and prostate [23], normalizing wound healing in diabetic rats [24], reduction in severity of periodontitis and inhibition of alveolar bone resorption associated with the disease in a rat model [25,26]. In addition, CMC2.24 has shown superior anti-inflammatory activity compared to the parent compound curcumin, in inhibiting periodontitis-induced bone resorption in rats via multiple mechanisms [27], and most recently, for the treatment of natural periodontitis in beagle dogs [28,29]. Other studies have established the pleotropic activities of CMC2.24 in inhibiting matrix metalloproteinases (MMPs) [30] and nuclear-factor kB (NF-kB), thereby reducing inflammation in models of diabetes-induced periodontitis [30]. Moreover, CMC2.24 as well as other CMCs—CMC2.14 and CMC2.23, have been shown to demonstrate chondroprotective effects in bovine cartilage explants [31]. Another triketonic curcumin derivative, CMC2.5 (4-methoxycarbonyl curcumin), has also been shown to possess activity superior to that of the parent compound (curcumin) in the treatment of inflammation in periodontitis [32]. The scheme for synthesis of these CMCs is based on the Pabon reaction and has been described previously [33].

Inspired by the key role of the β-diketone as a zinc-binding region [34], CMCs were initially developed as polyenolic zinc-binding inhibitors that share the β-diketone with antibiotic tetracyclines and non-antibiotic chemically modified tetracyclines (CMTs) that form a class of host-modulating agents (HMTs) developed by Golub et al. previously [34,35]. By virtue of their capacity to bind to zinc, these CMCs were capable of inhibiting MMPs and cytokines thus demonstrating anti-inflammatory efficacy. While natural curcumin is diketonic, a series of CMCs in the library that were originally synthesized consisted of diketonic as well as triketonic derivatives. Amongst these, the triketonic derivatives, in particular the lead compound, CMC2.24 exhibited enhanced acidic character of the enolic system due to its triketonic nature, resulting in a greater zinc-binding capacity and superior biological efficacy, that was much potent than curcumin and better than other diketonic CMCs. Hence, we selected CMC2.24 and similar triketonic derivatives (CMC2.23, CMC2.5 and CMC2.14) for analysis in the current study.

Here, we studied four CMCs: CMC2.14, CMC2.5, CMC2.23 and CMC2.24, which differ in (i) the type of substituent on the central carbon flanked by the β-diketone (methoxycarbonyl vs. phenylaminocarbonyl) and (ii) the presence or absence of a methoxy group on the aryl rings, for their capacity to inhibit tyrosinase enzyme activity and melanin production in B16F10 cells and further elucidate mechanism of inhibition. We have also included the parent compound, pure curcumin (PC; curcumin I) as a reference, which we have shown in our previous study to inhibit melanin levels in B16F10 cells [36]. Our working hypothesis is that of the four CMCs, CMC2.24 would possess the greatest inhibitory activity towards both steps of tyrosinase-catalyzed melanin production as well as the greatest diminution of cellular melanogenesis.

## 2. Materials and Methods

### 2.1. Materials

Pure curcumin (PC, 99% purity) was purchased from Selleck Chemicals (Houston, TX, USA). The chemically synthesized curcumin derivatives, CMC2.24, CMC2.5, CMC2.14 and CMC2.23 (all 97% purity) were obtained from Chem-Master International, Inc. (Hauppauge, NY, USA). Kojic Acid (KA), mushroom tyrosinase enzyme, α-glucosidase enzyme from Baker’s yeast, *p*-nitrophenyl β-d-glucopyranoside (pNG), l-DOPA, l-Tyrosine, copper sulfate and pyrocatechol violet (PV) were purchased from Sigma-Aldrich (St. Louis, MO, USA). Total protein levels were quantitated with a bicinchoninic acid (BCA) assay procured from ThermoFisher Scientific. 2,2-diphenyl-1-picrylhydrazyl (DPPH) reagent was purchased from Molecular Probes Inc. (Eugene, OR, USA). 

### 2.2. Monophenolase Activity Assay

The direct effects of the compounds on the monophenolase activity of tyrosinase were tested using mushroom tyrosinase enzyme with a fixed concentration of l-Tyrosine (l-Tyr) as the substrate. Briefly, 80 µL of compounds prepared at different concentrations in 50 mM sodium phosphate (pH 6.8) buffer were added to wells of a 96-well microplate followed by 100 µL of 2 mM l-Tyr solution. The reaction was initiated by adding 20 µL of mushroom tyrosinase (final concentration of enzyme in wells was 12.5 µg/mL) and was monitored by measuring absorbance at 475 nm every 30 s over a 30 min period, using a microplate reader (Versamax^®^) in the kinetic mode. The slopes of the plots of absorbance vs. time were calculated for comparison of enzyme activity in the presence of each of the inhibitors to that of an inhibitor-free control and reported as percentage of control activity (%).

### 2.3. Diphenolase Activity Assay

The direct effects of the compounds on the diphenolase activity of tyrosinase were tested using mushroom tyrosinase with a fixed concentration of l-DOPA as the substrate. Briefly, 80 µL of each compound prepared at different concentrations in 50 mM sodium phosphate buffer (pH 6.8) was added to wells of a 96-well microplate, followed by 100 µL of freshly prepared substrate solution (3 mM l-DOPA in buffer). The reaction was initiated by adding 20 µL of mushroom tyrosinase (final concentration of enzyme in wells was 3.5 µg/mL). The production of Dopachrome was monitored by measuring the absorbance at 475 nm every 30 s for a period of 30 min at 30 °C, using a microplate reader in the kinetic mode. The slopes of the plots of absorbance vs. time were calculated for comparison of enzyme activity in the presence of each of the inhibitors to that of the inhibitor-free control.

### 2.4. Kinetic Analysis of Monophenolase and Diphenolase Activity 

In order to study the mechanism of inhibition of tyrosinase activity by the compounds as a function of substrate concentration, we conducted a kinetic study of monophenolase and diphenolase activities at multiple concentrations of substrates. For the monophenolase activity assay, the final concentration of enzyme was 12.5 µg/mL and the final substrate concentrations were 0.125, 0.25, 0.5 and 1 mM, while for diphenolase activity assay, the final concentration of enzyme was 3.5 µg/mL and the final substrate concentrations ranged from 0.1–3 mM. The slopes from the linear range of the progress curves of absorbance at 475 nm vs. time were recorded as apparent velocities and the reciprocal values, 1/v, were plotted as functions of the reciprocal values of the substrate concentrations at different fixed inhibitor concentrations according to the method of Lineweaver and Burk (L-B). Dixon plots of the reciprocal velocity values, (1/v), as functions of the inhibitor concentrations at different fixed substrate concentrations were also constructed to investigate further the apparent mode of inhibition by the compounds.

### 2.5. Copper Chelating Assay

Copper chelation can be detected by a PV colorimetric assay as reported in other studies [37] and similar to the method reported in our previous study [38]. Briefly, 100 µL aliquots of different concentrations of compounds prepared in 50 mM sodium acetate buffer (pH 6.0) were added to a 96-well plate. The control group consisted of buffer only, while KA at 500 µM served as a positive control. Copper sulfate (2 mM; 10 µL) was added to each of the samples and incubated for 10 min. Next, 10 µL of 2 mM PV was added and the plate was further incubated for 20 min. The absorbance was read at 632 nm using a microplate reader; a lower absorbance compared to that of the buffer control was indicative of copper chelation.

### 2.6. Antioxidant Activity Assay

Melanin production catalyzed by the tyrosinase pathway is often associated with higher levels of oxidative stress and reactive oxygen species (ROS) generation [39]. Hence, compounds which possess antioxidant activity can also act as inhibitors of melanin production. DPPH (2,2-Diphenyl-1-picrylhydrazyl) is a stable free radical which changes color from purple to yellow upon reduction by antioxidant compounds. Briefly, 180 µL of DPPH reagent that was prepared in methanol was combined with 20 µL of different concentrations of the compounds in a 96-well plate to give a final DPPH concentration of 60 μM in each well. A control group consisted of DPPH only while ascorbic acid (AA) at 20 µM was used as a positive control. The plate was covered and incubated for 30 min, after which the absorbance was read at 517 nm and the DPPH radical scavenging activity was reported as % of control.

### 2.7. Cellular Assays

#### 2.7.1. Cell Viability

B16F10 mouse melanoma cells (CRL-6475™) were obtained from American Type Culture Collection (ATCC, Manassas, VA, USA) and cultured using Dulbecco’s Modified Eagle Medium (DMEM) supplemented with 10% heat-inactivated fetal bovine serum (HI-FBS) and 1% antibiotics (penicillin-streptomycin) in a humidified incubator with 95% air—5% CO_2_ at 37 °C. In order to test the four CMCs (CMC2.14, CMC2.24, CMC2.5 and CMC2.23) to that of PC, for their effects on melanin content, we first screened them for cytotoxicity using a tetrazolium assay with MTS (Promega CellTiter Aqueous One). Briefly, B16F10 cells were seeded at 4 × 10^3^ cells/well in a 96-well plate for 24 h. The compounds were diluted using culture medium to yield a final DMSO in all groups of 0.16% and added to the cell monolayers in wells after 24 h. Control was treated with 0.16% DMSO. At the end of 48 h, the medium was aspirated and replaced by 100 μL of fresh medium. 20 μL of MTS reagent was then added, and the plate was incubated at 37 °C for 40 min. At this point, the absorbance of 100 μL aliquots was read at 490 nm using a microplate reader. Cell viability was calculated from the absorbance values relative to control groups and expressed in %.

#### 2.7.2. Determination of Melanin Levels

B16F10 cells (1 × 10^5^ cells/well) were seeded in 12-well plates and cultured for 24 h. The medium was then replaced with compounds at nontoxic concentrations that were selected on the basis of the MTS assay and incubated for another 48 h. After the treatments, the cells were detached using TrypLE (1×), and cell pellets were washed in PBS. Cell pellets were visually observed for lightening of the pigment. After aspiration, 250 μL of 1N NaOH was added and heated to 70 °C to solubilize melanin. The aliquots were then transferred to a 96-well plate and absorbance was read at 475 nm using a microplate reader. The absorbance was normalized to total protein content and was expressed as % of control.

#### 2.7.3. Intracellular Tyrosinase Activity

B16F10 cells (4 × 10^4^ cells/well in 1 mL medium) were cultured in 24-well plates for 24 h. After that, the medium was replaced by fresh medium containing the compounds, and further incubated for 48 h. At the end of treatments, the cells were harvested, and cell pellets were washed in PBS and lysed and then centrifuged. Lysates (50 µL) were then aliquoted in a 96-well plate and 150 µL of 3 mM l-DOPA was added. The absorbance was measured at 475 nm every 30 s for 40 min at 30 °C using a microplate reader in the kinetic mode. The tyrosinase activity was calculated from the slope of the linear range of the velocity in the presence of each test compound and normalized to the total protein content and reported as % of control. 

#### 2.7.4. Recovery Study of Intracellular Tyrosinase Activity

In order to establish reversibility of tyrosinase inhibition by the CMCs, we conducted a recovery study for 48 h using the highest concentrations of the compounds that produced maximum inhibition of tyrosinase activity. B16F10 cells were plated in six-well plates at 3.5 × 10^4^ cells/well and then compounds were added the next day. Tyrosinase activity was estimated after 48 h exposure in cellular lysates and another set of cultures were continued with fresh medium without the compounds to study the recovery of tyrosinase activity for 48 h. Results are expressed as % tyrosinase activity for both 48 h exposure and 48 h recovery.

#### 2.7.5. Intracellular α-Glucosidase Assay

We next studied if compounds inhibited α-glucosidase activity in cell cultures. B16F10 cells (2.2 × 10^5^) were grown in 6-well plates and compounds were added next day for 48 h. Cells were harvested, lysed and 50 µL of lysates were aliquoted in a 96-well plate with 100 µL of 2 mM pNG substrate; rate of the formation of the yellow-colored reaction product, *p*-nitrophenol, was monitored at 405 nm for 30 min at 37 °C in a microplate reader (kinetic mode) and values were normalized to total protein contents. The cellular α-glucosidase activity was calculated as (rate of sample reaction/rate of control reaction) × 100 and was expressed as percentage of control. 

### 2.8. Cell-Free α-Glucosidase Assay

The effects of compounds on α-glucosidase were assayed based on the method decribed previously [40] with some modifications. 80 µL of samples (prepared in 0.05 M phosphate buffer, pH 6.8) were aliquoted in a 96-well microplate followed by the addition of 100 µL of 2.4 mM pNG substrate solution. A volume of 20 µL of 0.45 units of α-glucosidase enzyme from Baker’s yeast prepared in 0.05 M buffer was added to start the reaction; the rate of the formation of *p*-nitrophenol was monitored at 405 nm for 15 min at 37 °C in a microplate reader in kinetic mode. The enzyme activity (%) was calculated as: (rate of sample reaction/rate of control reaction) × 100%. 

### 2.9. MITF and cAMP Measurement

Microphthalmia Transcription Factor (MITF), a known transcription factor for the tyrosinase gene, is a master regulator of melanogenesis and is activated by cyclic adenosine monophosphate (cAMP) signaling after UV irradiation [41], reflecting the role of cAMP as a key maturation factor in melanogenesis [42]. The effects of PC and CMCs on MITF protein levels were assayed using a cell-based ELISA (Lifespan Biosciences, Seattle, WA, USA). Briefly, B16F10 cells were cultured in a 96- well plate at 1 × 10^4^ cells/well for 24 h and then the medium was replaced with fresh medium containing compounds in 0.1% DMSO and cultures maintained for further 48 h. The cells were then fixed, and subsequent steps were conducted according to the manufacturers’ instructions. MITF protein levels were normalized to cell density by measuring the absorbance of crystal violet stain added to the fixed cells and the data was reported as % of control. 

For assaying the levels of cAMP in B16F10 cellular lysates, a competitive ELISA (Enzo Life Sciences, Farmingdale, NY, USA) was used in non-acetylated format based on manufacturer instructions.

### 2.10. Statistical Analysis

One-way analysis of variance (ANOVA) with Tukey’s or Dunnett’s post hoc test was used, and all the analyses were conducted using GraphPad Prism software (version 8.0.0 for Windows, San Diego, CA, USA) and differences were considered statistically significant at *p* < 0.05. All data are reported as Mean ± SD.

## 3. Results

### 3.1. Effect of Compounds on Monophenolase Activity of Mushroom Tyrosinase

**Figure 1:**
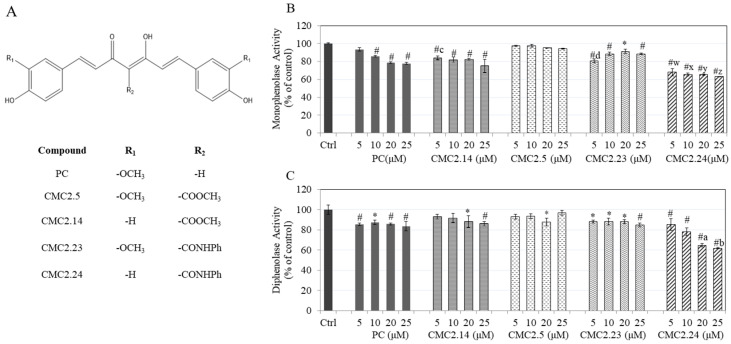
(**A**) Chemical structures of compounds obtained from modifications of the parent structure of curcumin. R_1_ denotes group modification of the two phenyl rings (-OCH_3_/-H) and R_2_ denotes group modification of the β-diketone moiety (-CONHPh/-COOCH_3_); Effect of the compounds on (**B**) Monophenolase activity and; (**C**) Diphenolase activity of mushroom tyrosinase; Data are mean ± SD of triplicates; * *p* < 0.01 and # *p* < 0.001 vs. Ctrl, letter c—*p* < 0.001 vs. PC (5 μM) and letter d—*p* < 0.001 vs. PC (5 μM); Letters-w, x, y, z all *p* < 0.001 vs. PC at 5, 10, 20, 25 μM, respectively; letter a—*p* < 0.001 vs. PC (20 μM) and letter b—*p* < 0.001 vs. PC at 25 μM; One-way ANOVA followed by Tukey’s test.

The chemical structure of PC and the chemical modification of the substituents on its ring to give the four analogs are illustrated in Figure 1A. Our results on the inhibition of monophenolase activity of tyrosinase showed that PC significantly inhibited monophenolase activity by 14.41%, 21.31%, and 22.10% at concentrations of 10, 20, and 25 μM, respectively. CMC2.24 inhibited the monophenolase activity by 32.08%, 34.66%, 34.84%, and 37.02% at 5, 10, 20, and 25 μM, respectively; this inhibition was significantly different from control as well as significantly greater than that of equivalent concentrations of the parent compound PC (Figure 1B). The other three CMCs exhibited moderate inhibitory activity which was similar across all the inhibitor concentrations tested. Taken together, CMC2.24 exhibited the greatest inhibitory activity of all the CMCs, with a significant inhibition at all tested concentrations. 

### 3.2. Effect of Compounds on Diphenolase Activity of Mushroom Tyrosinase

Our results of inhibition of diphenolase activity showed that PC significantly inhibited diphenolase activity by 14.47%, 12.41%, 14.19%, and 16.39% at concentrations of 5, 10, 20, and 25 μM, respectively. CMC2.24 inhibited diphenolase activity by 35.39% at 20 μM and 38.2% at 25 μM, which was significantly greater than that achieved by corresponding concentrations of PC (Figure 1C). The other CMCs (CMC2.14, CMC2.5, CMC2.23) exhibited somewhat weaker inhibitory potency, likely attributable to their specific chemical modifications. Altogether, our results of diphenolase activity by the compounds revealed a similar trend as that of our earlier results of monophenolase activity with CMC2.24 emerging as the best candidate as a tyrosinase inhibitor. 

The IC_50_ values for monophenolase and diphenolase inhibition by compounds, PC and CMC2.24, at fixed substrate concentrations were calculated and compared with those for kojic acid (KA), a well-known tyrosinase inhibitor; the results are summarized in Table S1. CMC2.24 had an IC_50_ value of 25.05 ± 1.18 µM which was 2.67-fold lower than PC (IC_50_: 66.99 ± 2.40 µM) and similar to KA (IC_50_: 23.93 ± 0.96 µM) for inhibition of monophenolase activity. The dose-response plots for monophenolase and diphenolase activity inhibition for compounds are shown in supplementary material (Figure S1). For the inhibition of diphenolase activity, CMC2.24 had an IC_50_ of 37.17 ± 3.70 µM which was 8-fold lower than PC and 1.9-fold lower than KA (IC_50_: 70.87 ± 6.10 µM). Overall, the results showed that CMC2.24 was significantly more potent than PC as a tyrosinase inhibitor for both substrates; its anti-monophenolase activity was similar to that of KA and its anti-diphenolase activity was far better than that of KA.

### 3.3. Evaluation of Mechanism of Inhibition Using Linear L-B and Dixon Plots

As CMC2.24 demonstrated the greatest inhibition of both the monophenolase and diphenolase activities of tyrosinase, we assayed it further, along with PC to elucidate the mechanisms of action. CMC2.23 was also included for comparison, as it was closely related to CMC2.24 structurally. To this end, we analyzed the inhibition by PC, CMC2.24, and CMC2.23 of monophenolase activity and diphenolase activity as a function of substrate concentrations at a series of fixed inhibitor concentrations by the “double reciprocal plot” method of L-B, in which values of 1/v are plotted as functions of 1/S at different fixed inhibitor concentrations. These plots generate a family of straight lines whose intersection can indicate the type of inhibition (competitive, uncompetitive, or noncompetitive). 

**Figure 2:**
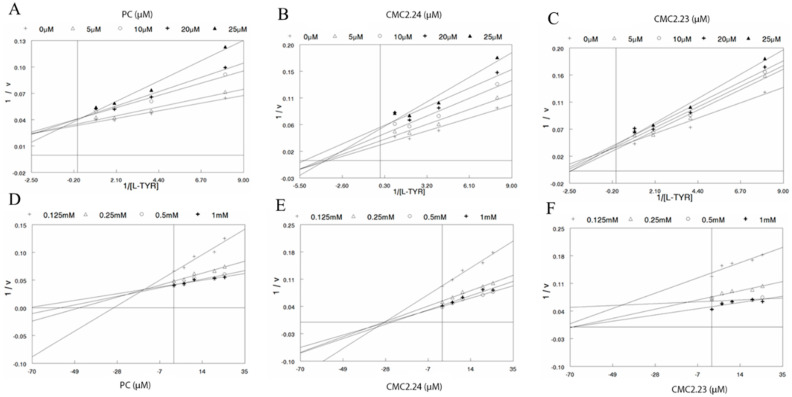
Kinetic analysis of monophenolase inhibition by PC, CMC2.24 and CMC2.23; Lineweaver-Burk plots showing the reciprocal of velocity (1/v) of tyrosinase reaction vs. reciprocal of substrate (1/l-TYR) for (**A**) PC; (**B**) CMC2.24 and; (**C**) CMC2.23; Dixon plots for the inhibition of monophenolase activity of mushroom tyrosinase by (**D**) PC; (**E**) CMC2.24 and; (**F**) CMC2.23 in the presence of different concentrations of l-TYR substrate (0.125, 0.25, 0.5 and 1 mM).

For the inhibition of monophenolase activity, analysis of the L-B plot (Figure 2A) and Dixon plot (Figure 2D) showed that PC is not a pure competitive inhibitor since all the lines did not intersect on the *y*-axis and the apparent value of Vmax changed with inhibitor concentration. Moreover, the mechanism was also not that of pure noncompetitive inhibition, since all lines should have intersected on the *x*-axis in the presence of a pure noncompetitive inhibitor, while the lines should have been parallel to each other for the mechanism to be that of pure uncompetitive inhibition. A similar mechanism of mixed inhibition was observed for CMC2.24 based on the L-B (Figure 2B) and Dixon plots (Figure 2E) in the presence of fixed concentrations of inhibitor or substrate. Moreover, the analysis of the mechanism of inhibition for CMC2.23 by L-B (Figure 2C) and Dixon plot (Figure 2F) showed that, similar to CMC2.24, CMC2.23 also acts through a mixed mode of inhibition.

**Figure 3:**
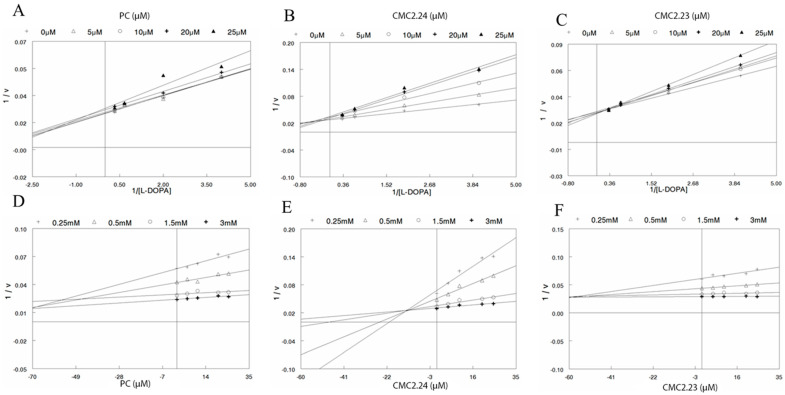
Kinetic analysis of diphenolase inhibition by PC, CMC2.24 and CMC2.23. Lineweaver-Burk plots showing the reciprocal of velocity (1/v) of tyrosinase reaction vs. reciprocal of substrate (1/l-DOPA) for (**A**) PC; (**B**) CMC2.23 and; (**C**) CMC2.23; Dixon plots for the inhibition of diphenolase activity of mushroom tyrosinase by (**D**) PC; (**E**) CMC2.24 and; (**F**) CMC2.23 in presence different concentrations of l-DOPA substrate (0.25, 0.5, 1.5 and 3 mM).

For the inhibition of diphenolase activity, analysis of the L-B plot (Figure 3A) and Dixon plot (Figure 3D) for PC showed that, as was the case for inhibition of the monophenolase reaction, PC is not a pure competitive inhibitor, but the mechanism was also not that of pure noncompetitive inhibition, or pure uncompetitive inhibition. A similar mixed mechanism of inhibition was observed again for CMC2.24, based on analysis of L-B (Figure 3B) and Dixon plot (Figure 3E) as well as CMC2.23 based on its L-B (Figure 3C) and Dixon plot (Figure 3F).

### 3.4. Mechanism of Inhibition Using Non-Linear Curve Fitting

We have also analyzed the inhibition data using nonlinear curve fitting algorithms which are more robust than the linearized L-B and Dixon plotting methods that can weigh the data from evenly spaced concentrations disproportionately. The inhibition constants were calculated by non-linear least squares curve fitting using a modified Levenberg-Marquardt algorithm and data was fitted to models of pure competitive, noncompetitive, and uncompetitive inhibition using Enzfitter software (version 2.0, Biosoft, Cambridge, UK). This analysis was undertaken to assess the apparent potency of compounds, PC, CMC2.24, and CMC2.23 for each putative mode of inhibition. Analysis using this algorithm showed that for the case of monophenolase inhibition, data for PC could be fit to a model of pure competitive inhibition with an apparent Ki of 7.74 μM, while the fits to other modes of inhibition suggested significantly weaker inhibitory potency. A fit of the data for PC to a model of pure noncompetitive inhibition gave an apparent Ki of 50.76 μM and a fit to pure uncompetitive inhibition gave an apparent Ki of 40.2 μM which was 1.26 times lower than that of pure noncompetitive inhibition (Table S2), indicating that PC is bound with greatest affinity to the enzyme as a competitive inhibitor for l-TYR. For inhibition of diphenolase activity, curve-fitting to a model of pure competitive inhibition yielded an apparent Ki of 45.15 μM for PC, while the fits to other modes of inhibition suggested significantly weaker inhibitory potency. A fit of the data for PC to a model of pure noncompetitive inhibition gave an apparent Ki of 163.7 μM and a fit to a model of pure uncompetitive inhibition gave an apparent Ki of 129 μM, which was 0.79 times lower than that of pure uncompetitive inhibition (Table S2), indicating that PC is bound with greatest affinity to the enzyme as a competitive inhibitor of l-DOPA. PC thus appears to be a mixed inhibitor of both the monophenolase and diphenolase activities of tyrosinase that displays a predominantly competitive mechanism with significantly weaker contributions of noncompetitive and uncompetitive mechanisms. The computed values of the apparent Km for monophenolase activity, using l-TYR as substrate were lower than the computed Km values for diphenolase activity, using l-DOPA as substrate (Table S2), which indicated that enzyme generally had greater affinity for the monophenolase substrate than the diphenolase substrate.

The inhibition mechanism was next studied for CMC2.24, which showed a mixed mode of inhibition with a predominantly competitive mechanism, similar to PC. Curve-fitting using l-TYR as substrate gave an apparent value of Ki of 4.56 µM for CMC2.24 as a competitive inhibitor of monophenolase activity, while a fit to pure uncompetitive inhibition gave an apparent Ki value of 23.64 μM and a fit to pure noncompetitive inhibition gave an apparent Ki value of 16.47 μM, which was 1.44 times lower than that for the pure uncompetitive mode (Table S2). For inhibition of diphenolase activity, curve-fitting to a model of pure competitive inhibition gave an apparent Ki value for CMC2.24 of 7.83 μM, while a fit to a model of pure uncompetitive inhibition gave an apparent Ki value for CMC2.24 of 32.54 μM and a fit to a model of pure noncompetitive inhibition gave an apparent Ki value of 42.11 μM (Table S2). The mechanism of inhibition of compound CMC2.23 was also studied by curve-fitting (Table S2); it also exhibited a similar mixed, but predominantly competitive, mode of inhibition of the monophenolase and diphenolase activities of mushroom tyrosinase.

Collectively, these data indicate that PC as well as CMC2.24 and CMC2.23 may be capable of binding both to the free enzyme and, with significantly lower affinity, to the enzyme-substrate complex (ES complex).

### 3.5. Effect of Compounds on Copper Chelation

**Figure 4:**
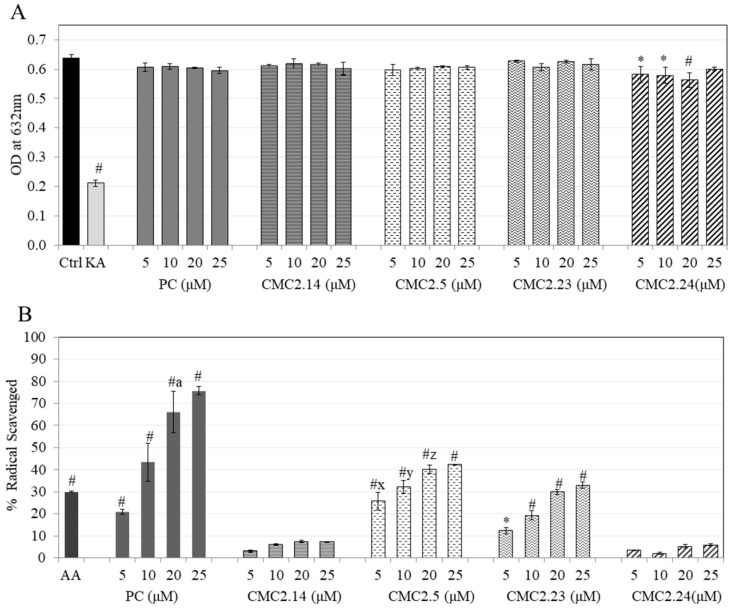
(**A**) Copper chelating activity indicated by reduction in absorbance at 632 nm was tested for compounds; * *p* < 0.01 and #-*p* < 0.001 vs. Ctrl; One-way ANOVA followed by Tukey’s test; (**B**) DPPH radical antioxidant activity indicated by % radical scavenged for compounds; * *p* < 0.01 and # *p* < 0.001 vs. control (baseline considered as 0% scavenged); Letter a—*p* < 0.001 vs. positive control AA (20 μM); Letter x—*p* < 0.001 vs. CMC2.23 at 5 μM; Letter y—*p* < 0.01 vs. CMC2.23 at 10 μM; Letter z—*p* < 0.05 vs. CMC2.23 at 20 μM; Data are mean ± SD (n = 3 per group); One-way ANOVA followed by Tukey’s test.

As tyrosinase is a binuclear copper enzyme, we further studied if the compounds inhibited tyrosinase via copper chelation, for which the PV dye method was used. CMC2.24 at 20 µM showed a significant copper chelating activity of 11.86% compared to the control, while all the other CMCs and PC did not show any effect on copper chelation (Figure 4A). 

### 3.6. Effect of Compounds on Antioxidant Activity via DPPH Assay

The results for DPPH radical scavenging activity of the compounds are summarized in Figure 4B. As expected, PC showed high radical scavenging activity, while CMC2.14 and CMC2.24 did not demonstrate such activity. However, the compounds CMC2.5 and CMC2.23 showed significant radical scavenging activity with CMC2.5 showing the highest scavenging activity amongst all the CMCs tested. Overall, the order of potency was PC > CMC2.5 > CMC2.23 > CMC2.14 = CMC2.24.

### 3.7. Effect of Compounds on Cytotoxicity in B16F10 Cells

**Figure 5:**
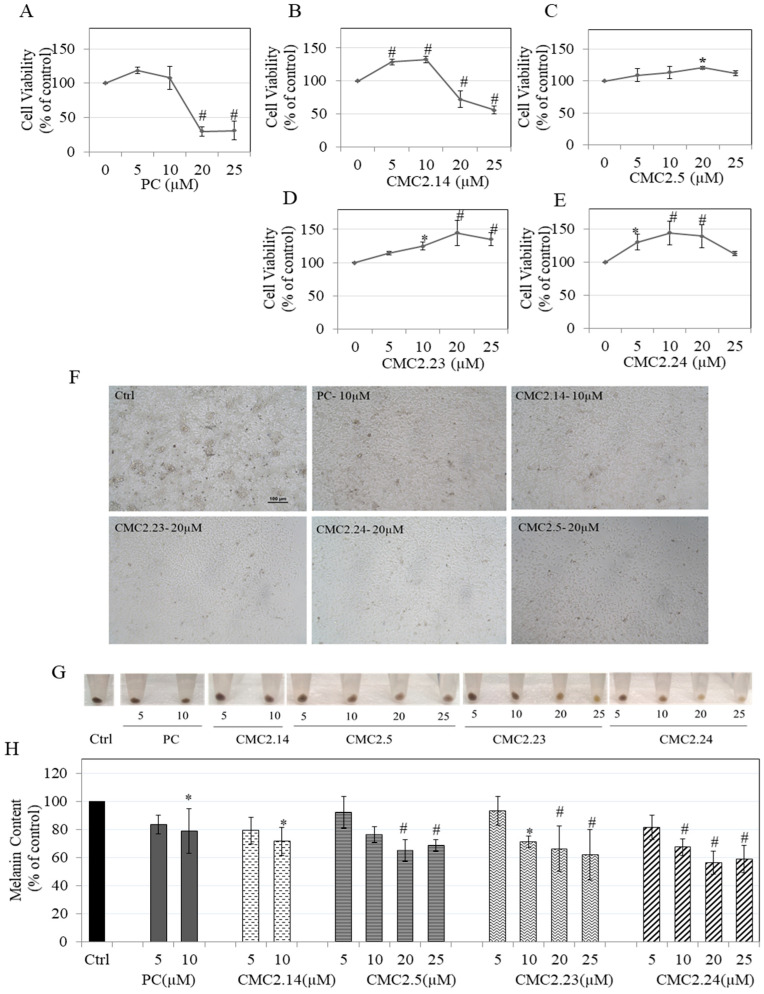
Viability of B16F10 cells treated for 48 h in the presence of different concentrations of PC and CMCs; (**A**) PC; (**B**) CMC2.14; (**C**) CMC2.5; (**D**) CMC2.23 and; (**E**) CMC2.24, measured using MTS cytotoxicity assay. * *p* < 0.05 and # *p* < 0.01 vs. control; (**F**) Representative images of B16F10 cells showing the control group and treatment groups (PC and CMC2.14 (10 µM), CMC2.5, CMC2.23 and CMC2.24 (20 µM)); Melanin content estimation with different concentrations of PC and CMCs showing (**G**) Cell pellet panel shows representative images from one experiment and; (**H**) quantification of relative melanin levels expressed as % of control. Control was treated with 0.16% DMSO. * *p* < 0.01 and # *p* < 0.01 vs. control; One-way ANOVA with Dunnett’s post hoc test; All data are mean ± SD of at least three independent experiments.

MTS assay was conducted to screen the compounds for cytotoxicity in order to evaluate nontoxic concentrations of the compounds for their effects on melanogenesis. Both PC and CMC2.14 induced significant cytotoxicity at 20 and 25 µM. The mean values of cell viability were 29.47% and 30.83% in the presence of 20 and 25 µM PC, respectively (Figure 5A), while the cell viability was measured to be 72.22% and 55.54% in the presence of 20 and 25 µM CMC2.14 (Figure 5B). CMC2.5 (Figure 5C), CMC2.23 (Figure 5D), and CMC2.24 (Figure 5E) were nontoxic over the full concentration range. All the compounds appeared to induce an increase in reduction of the tetrazolium salt MTS to formazan at lower concentrations; CMC2.24 showed the maximal capacity to reduce MTS to its formazan, out of the four derivatives. Overall, PC was found to be most cytotoxic. Based on these results, we used compounds PC and CMC2.14 over a concentration range 5–10 µM and compounds CMC2.5, CMC2.24, and CMC2.23 over the range 5–25 µM for subsequent experiments.

### 3.8. Effect of Compounds on Melanin Levels in B16F10 Cells

The photomicrographs of cells treated with different compounds showed higher number of melanin granules in cells in control group which were visibly diminished in CMC-treated groups (Figure 5F). The panel (Figure 5G) showed that the pellets of cells treated with CMC2.23 and CMC2.24 displayed a visibly lighter appearance compared to the other CMCs at higher concentrations. Our results of the quantitation of melanin content showed that PC at 10 µM significantly suppressed melanin levels by 20.92%, which was similar to the levels of suppression obtained by CMC2.14 at 10 µM (28.42%). CMC2.5 significantly suppressed melanin levels by 34.84% and 31.40% only at higher concentrations of 20 and 25 µM, respectively (Figure 5H). CMC2.23 showed a significant reduction in melanin levels by 28.63%, 33.68%, and 37.87% at 10, 20, and 25 µM, respectively, while CMC2.24 showed a significant reduction in melanin levels by 32.54%, 43.53%, and 41.07% at 10, 20, and 25 µM, respectively. 

Collectively, these results suggest that while PC showed a moderate efficacy (with cytotoxicity at concentrations > 10µM), a greater efficacy with 1.5-fold and 2-fold higher levels of melanin inhibition could be achieved by CMC2.24 at concentration of 20 and 25 µM, respectively; this inhibitory efficacy was considerably greater than that achieved with CMC2.14 as well as CMC2.5, while it was somewhat better than that achieved with CMC2.23, although the profile of inhibition was similar. 

### 3.9. Effect of Compounds on Intracellular Tyrosinase Activity in B16F10 Cells

**Figure 6:**
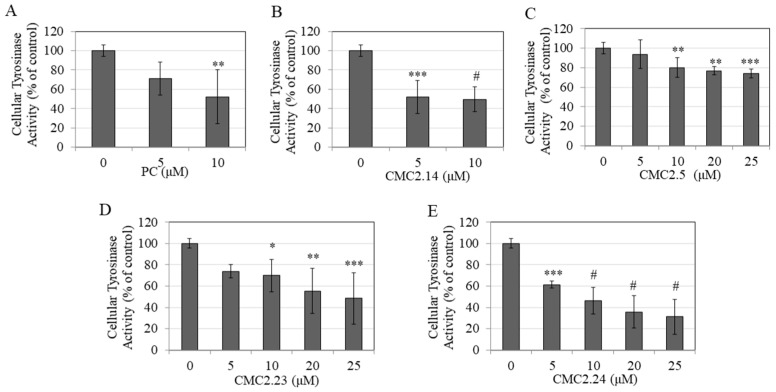
Tyrosinase activity in B16F10 cells treated for 48 h with different concentrations of compounds; (**A**) PC; (**B**) CMC2.14; (**C**) CMC2.5; (**D**) CMC2.23 and; (**E**) CMC2.24. KA (500 μM) was used as a positive control. One-way ANOVA and Dunnett’s post hoc test; * *p* < 0.05; ** p < 0.01; *** *p* < 0.001; and # *p* < 0.0001 vs. control. One-way ANOVA with Dunnett’s post hoc test. All data are mean ± SD of values pooled from two independent experiments.

To identify the mechanism of depigmentation, we evaluated tyrosinase activity in B16F10 cellular lysates after treatment with the compounds. PC showed a significant suppression of tyrosinase activity by 47.85% at 10 µM (Figure 6A). 

CMC2.14 significantly suppressed tyrosinase activity by 47.96% and 50.31% at 5 and 10 µM, respectively (Figure 6B), while CMC 2.5 showed a significant suppression of tyrosinase activity by 19.98%, 23.16%, and 26% at 10, 20, and 25 µM, respectively (Figure 6C). CMC2.23 significantly suppressed tyrosinase activity by 30.04%, 44.64%, and 51.47% at 10, 20, and 25 µM, respectively (Figure 6D), while CMC2.24 significantly suppressed tyrosinase activity by 38.6%, 53.76%, 64.25%, and 68.81% at 5, 10, 20, and 25 µM, respectively (Figure 6E). Taken together, these results demonstrate that CMCs inhibit melanogenesis, at least in part, by inhibiting intracellular tyrosinase activity where CMC2.24 and CMC2.14 showed the greatest inhibitory effect while CMC2.5 showed weakest effect on tyrosinase activity.

### 3.10. Recovery Study of Tyrosinase Activity in B16F10 Cells

**Figure 7:**
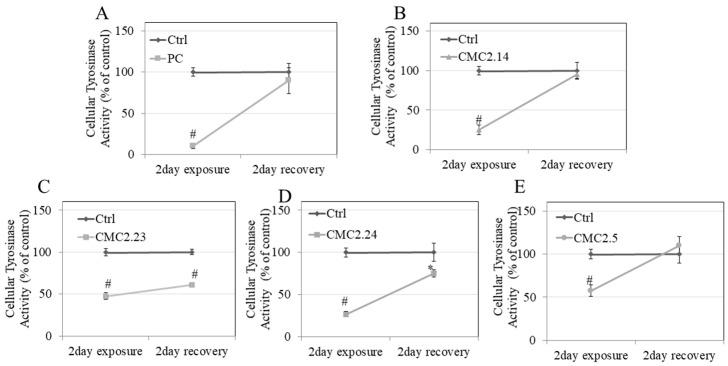
Exposure and recovery study of tyrosinase activity in lysates of B16F10 cells treated with compounds, (**A**) PC (10 µM); (**B**) CMC2.14 (10 µM); (**C**) CMC2.23 (20 µM); (**D**) CMC2.24 (20 µM), and (**E**) CMC2.5 (20 µM); # *p* < 0.01 vs. control at 2-day exposure; # *p* < 0.01 vs. control at 2-day exposure; * *p* < 0.05 vs. control at 2 day recovery; All data are mean ± SD (*n* = 3 per group).

The results on apparent recovery of intracellular tyrosinase activity showed that treatment with PC at 10 µM robustly suppressed tyrosinase activity in B16F10 cells by 89.52% which was rapidly recovered by the cells after removal of PC to the extent that the activity of cells exposed to PC (which was then removed) did not differ from that of the control group (Figure 7A). Next, we exposed the cells to CMC2.14 at 10 µM, and the other three CMCs (CMC2.24, CMC2.23, and CMC2.5) at 20 µM, followed by replacement with compound-free medium. CMC2.14 at 10 µM caused a loss of 74.81% of tyrosinase activity which was rapidly recovered to 100% after replacement with compound-free medium (Figure 7B). CMC2.23 at 20 µM caused a significant loss of tyrosinase activity by 52.67% which was only partially recovered as the tyrosinase activity was still significantly lower than the recovery control by 38.78% (Figure 7C). A similar profile was noted for CMC2.24 at 20 µM which significantly suppressed tyrosinase activity by 73.27% that was partially recovered (albeit to a higher degree as compared to CMC2.23) with the activity still significantly lower than the recovery control group by 24.62% (Figure 7D). CMC2.5 at 20 µM caused a loss of 42.54% of tyrosinase activity which was rapidly recovered by the cells to 100% (Figure 7E). Altogether, our results show that after exposure of the cells to three CMCs (CMC2.23, CMC2.24 and CMC2.5) at similar concentrations, followed by their continued culture in CMC-free medium, only cells exposed to CMC2.5 could fully recover their tyrosinase activity while longer recovery periods may be needed for the intracellular tyrosinase activity to be fully restored after exposure to CMC2.23 and CMC2.24. 

### 3.11. Effect of Compounds on α-Glucosidase Enzymatic Activity in B16F10 Cells

**Figure 8:**
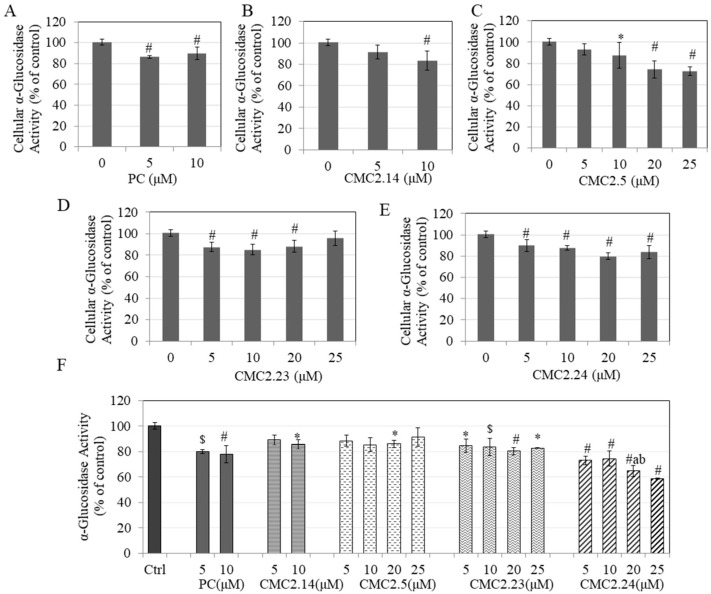
α-glucosidase activity in B16F10 cells treated for 48 h with different concentrations of compounds; (**A**) PC; (**B**) CMC2.14; (**C**) CMC2.5; (**D**) CMC2.23 and; (**E**) CMC2.24; One-way ANOVA with Dunnett’s post hoc test. * *p* < 0.05 and # *p* < 0.01 vs. control; Data are combined from at least two independent experiments; (**F**) α-glucosidase activity in cell-free system with different concentrations of PC and CMCs measured using pNG substrate. * *p* < 0.05; $ *p* < 0.01 and # *p* < 0.001 vs. control; letter a—*p* < 0.05 vs. CMC2.23 (20 µM); letter b—*p* < 0.001 vs. CMC2.5 (20 µM); One-way ANOVA with Tukey’s test; Data are mean ± SD of triplicate determinations.

Next, we measured the levels of α-glucosidase activity in B16F10 cellular lysates after treatment with the compounds to evaluate whether these compounds might also inhibit the enzyme activity at the cellular level. PC significantly inhibited the intracellular enzyme activity by 13.71% and 10.47% at concentrations of 5 and 10 µM, respectively (Figure 8A), while CMC2.14 significantly inhibited the activity by 16.49% at concentration of 10 µM (Figure 8B). CMC2.5 demonstrated a potent inhibition of enzyme activity which was significant at all concentrations >5 µM; an inhibition of 12.45%, 25.58% and 27.38% was achieved at concentrations of 10, 20, and 25 µM, respectively (Figure 8C). CMC2.23 significantly inhibited cellular α-glucosidase activity by 12.45% 14.94% and 11.84% at 5, 10 and 20 µM, respectively, with no change at 25 µM (Figure 8D). Lastly, CMC2.24 significantly inhibited cellular α-glucosidase activity at all concentrations; levels of inhibition of 10.22%, 12.61%, 20.31%, and 16.36% were achieved at concentrations 5, 10, 20, and 25 µM, respectively (Figure 8E). CMC2.23 did not inhibit cellular α-glucosidase activity even at 25 µM, whereas CMC2.5 significantly inhibited the cellular activity. Overall, our results showed that all the CMCs including PC exhibit a capacity to suppress the α-glucosidase activity at cellular level, which indicates that the mechanism of melanin inhibition of CMCs is attributable, at least in part, to their capacity to inhibit tyrosinase maturation by interfering with *N*-glycan processing.

### 3.12. Effect of Compounds on Cell-Free α-Glucosidase Enzymatic Activity

PC significantly inhibited α-glucosidase activity in vitro by 20.13% and 22.13% at concentrations of 5 and 10 µM, respectively, while CMC2.14 showed significant inhibition by 14.34% only at concentration of 10 µM (Figure 8F). CMC2.5 only showed a significant inhibition by 14.04% only at 20 µM, while no change was observed at any other concentration (Figure 8F). CMC2.23 suppressed the α-glucosidase activity significantly at all concentrations; an inhibition of 15.49%, 16.47%, 19.82%, and 17.25% was obtained at concentrations of 5, 10, 20, and 25 µM, respectively (Figure 8F). On the other hand, CMC2.24 significantly suppressed the α-glucosidase activity in a concentration-dependent manner at all the tested concentrations; an inhibition of 27.07%, 25.58%, 35.33%, and 41.33% were obtained at concentrations of 5, 10, 20, and 25 µM, respectively (Figure 8F). Altogether, these results reveal that CMC2.24 was the most potent CMC analog amongst all the other analogs to inhibit in vitro α-glucosidase activity; it was also far more potent than PC. The results further demonstrate that CMC2.24 has a pronounced effect in inhibiting the α-glucosidase activity; they suggest a direct inhibitory effect on early *N*-glycan processing and on maturation of tyrosinase in the Golgi apparatus. 

### 3.13. Effect of Compounds on cAMP Levels inB16F10 Cells

**Figure 9:**
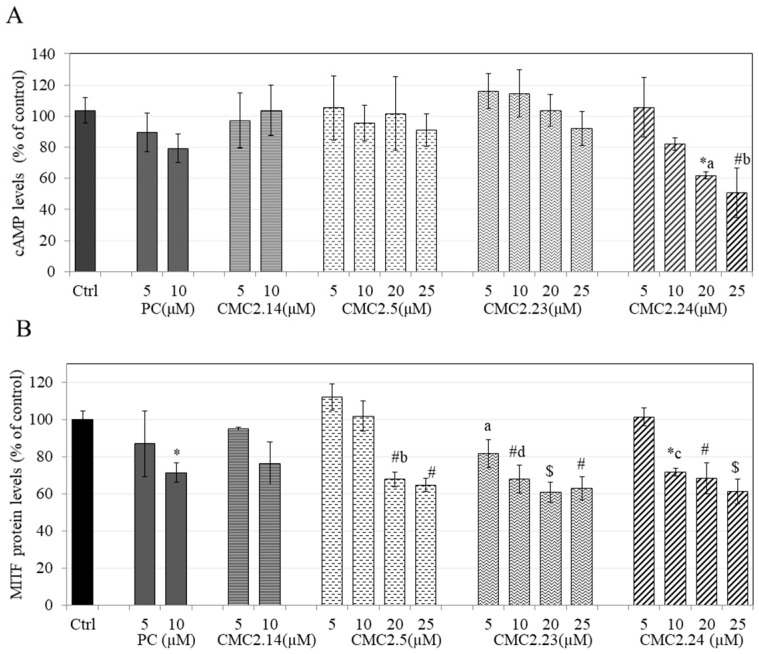
(**A**) cAMP levels estimated by ELISA in cultures of B16F10 cells treated for 48 h with different concentrations of PC and CMCs, * *p* < 0.05 and # *p* < 0.01 vs. control; letter a—*p* < 0.05 vs. CMC2.5 and CMC2.23 at 20 µM; letter b—*p* < 0.05 vs. CMC2.5 and CMC2.23 at 25 µM; One-way ANOVA with Tukey’s post hoc test; Data are mean ± SD of at least two independent experiments; (**B**) MITF protein levels estimated by cell-based ELISA in cultures of B16F10 cells treated for 48 h with different concentrations of PC and CMCs, * *p* < 0.01; # *p* < 0.01 and $ *p* < 0.001 vs. control; letter a—*p* < 0.05 vs. CMC2.5 (5 µM); letter b—*p* < 0.01 vs. CMC2.5 (10 µM); letter d—*p* < 0.01 vs. CMC2.5 (10 µM); letter c—*p* < 0.05 vs. CMC2.24 (5 µM); One-way ANOVA with Tukey’s test; Data are mean ± SD (*n* = 3 per group).

Our results showed that CMC2.24 at 10 µM attenuated cAMP levels by 18.13% but levels were not significant, while higher concentrations of 20 µM and 25 µM significantly attenuated cAMP levels by 38.18% and by 49.42%, respectively. PC showed a trend for attenuation of cAMP levels, but the levels were not significant (Figure 9A). These results indicate that only CMC2.24 showed a significant attenuation of cAMP levels in a concentration-dependent manner, while the other three analogs, CMC2.14, CMC2.5, and CMC2.23 did not affect cAMP levels at any concentration. Altogether, these data suggest that the anti-melanogenic mechanism of CMC2.24 involves the downregulation of cAMP levels, while the other CMCs inhibit melanogenesis by mechanisms not involving cAMP downregulation. 

### 3.14. Effect of Compounds on MITF Protein Levels in B16F10 Cells

The results of the effects of compounds on MITF protein level in B16F10 cells is summarized in Figure 9B. CMC2.24 significantly attenuated MITF protein levels by 28.39%, 31.79% and 38.84% at 10, 20 and 25 µM, respectively. The attenuation of MITF levels by CMC2.24 at 10 µM was also significant as compared to attenuation of MITF protein levels by CMC2.24 at 5 µM. CMC2.23 significantly diminished MITF protein levels by 32.27%, 39.29% and 37.22% at 10, 20 and 25 µM, respectively. CMC2.5 significantly diminished MITF levels by 32.17% at 20 µM and by 35.4% at 25 µM. The diminution of MITF levels by CMC2.5 at higher concentration of 20 µM was also significant as compared to CMC2.5 at 10 µM. PC at 10 µM significantly diminished MITF protein levels by 28.62% while CMC2.14 at 10 µM reduced the protein levels by 23.67% but the difference in levels did not reach significance.

Overall, all the CMCs (except CMC2.14) significantly diminished MITF protein levels; CMC2.23 appeared to be somewhat more potent than CMC2.5 at lower concentrations of 5 µM and 10 µM, while at higher concentrations of 20 µM and 25 µM, all the three CMCs (2.24, 2.5 and 2.23) showed similar inhibitory profiles, and the levels of inhibition were not different from each other.

## 4. Discussion

In the current study, we have evaluated four CMC analogs and compared them to pure curcumin (PC); we have established their efficacies as inhibitors of the in vitro activities of mushroom tyrosinase as well as their inhibition of cellular melanogenesis. The superior zinc-chelating ability of these curcumin analogs [43] had initially prompted us to study the unique metal-binding capacity of these derivatives in the context of inhibition of tyrosinase, which has two copper atoms in its structure. Interestingly, a recent study has demonstrated the presence of two zinc atoms instead of two copper atoms in the active site of the tyrosinase-like subdomain of tyrosinase-related protein 1 (TYR-1), which is similar to tyrosinase [44]. We have previously reported [21] the inhibition of the zinc metalloprotease, lethal factor, by curcumin and curcumin derivatives in which a mixed, but predominantly competitive mode of inhibition was observed for both CMC2.24 and PC. PC was found to be better than CMC2.24 at inhibition of lethal factor, but the inferior stability and solubility of the former hindered its potential for further use. Our results in this study showed that CMC2.24 was the most potent tyrosinase inhibitor and showed better efficacy than PC as an inhibitor of monophenolase (IC_50_- 25 µM) and diphenolase activities (IC_50_-37 µM). In addition, CMC2.24 showed an IC_50_ against the monophenolase activity of tyrosinase comparable to that of the established tyrosinase inhibitor, kojic acid (KA) and a 1.9-fold lower IC_50_ than KA against the diphenolase activity of the enzyme (Table S1). Our results demonstrate that the CMCs were predominantly competitive inhibitors of the diphenolase activity of tyrosinase, similar to PC. We have used tyrosinase purified from mushrooms as the enzyme source in assays in which compounds were tested for their direct effect on cell-free enzyme activity due to its wide availability and its validation in several studies, although there are limitations associated with its use. First, mushroom tyrosinase is a monomer while human tyrosinase is a tetramer which can undergo glycosylation and maturation [45]. Second, mushroom tyrosinase is a soluble cytosolic enzyme while human tyrosinase is membrane-bound [46]. A recent report has also established that some inhibitors of mushroom tyrosinase do not necessarily inhibit human tyrosinase as the latter has different molecular motifs [47]. Previously, a large library of chemically synthesized curcumin derivatives has been tested by another group for their capacity to inhibit tyrosinase [48]. However, due to a lack of any information on their solubility and stability in addition to scarcity of their safety and toxicological studies, the practical applications of these analogs in inhibiting melanogenesis in cell cultures cannot be confirmed. Although the SAR studies were conducted on a limited library of CMCs, we have selected these analogs from the initial library of several CMCs on the basis of their established biological efficacies; our limited SAR may offer a lead for the potential synthesis of newer effective tyrosinase inhibitors in which the β-diketone is replaced with a triketone. 

**Figure 10:**
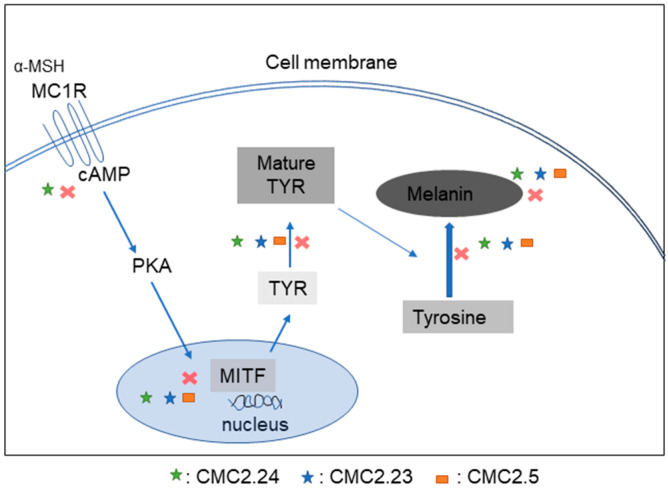
Proposed scheme showing the different targets at which CMCs inhibit the steps of melanogenesis in B16F10 cells.

Our results of significant antioxidant activity for compounds CMC2.23 and CMC2.5, both of which possess a methoxy group on each of the two aryl rings, is indicative of the importance of this moiety in DPPH antioxidant activity. This result agrees with a previous study in which carbocyclic curcumin analogs were studied; the authors noted that the electron-withdrawing moiety compromised the analogs’ antioxidant activity [49]. Our results in cellular assays showed that PC was less effective at inhibiting melanin production in B16F10 melanoma cells as compared to CMCs with a significant cytotoxicity at concentrations >10 µM. A previous study has also reported that curcumin was highly cytotoxic to breast cancer cells at concentrations >10 µM [50]. Our results in cellular assays further demonstrated that all the CMCs were effective melanogenesis inhibitors and appeared to interfere with multiple steps in the melanogenesis pathway via pleotropic modes of action. The various steps which are targeted by the three CMCs by their pleiotropic mode are summarized in a scheme (Figure 10).

UV irradiation is known to produce α-MSH, a pro-melanogenic stimulus, and there have been reports in which curcumin inhibited melanin levels under αMSH-stimulated conditions [51,52]. Our results demonstrate that CMCs exhibited potent inhibition of melanogenesis even under αMSH-stimulated conditions (Figure S2), which confirms their utility as anti-melanogenic agents under both spontaneous and hormone-stimulated conditions. 

Normal melanin production is necessary for photoprotection and immune regulation, and since tyrosinase is one of the key enzymes in melanogenesis pathway, skin-lighteners which cause irreversible inhibition of tyrosinase activity can pose safety concerns. Previous studies have reported the recovery of tyrosinase activity in melanocytes by anti-melanogenic compounds [53,54]. Hence, we have evaluated CMCs for reversibility of their inhibition of tyrosinase activity using the highest concentrations for each of the CMCs. To the best of our knowledge, this is the first study to test recovery of tyrosinase activity by curcumin and its derivatives. Our results on recovery study of tyrosinase activity showed that cells treated with CMC2.14 (10 µM) or CMC2.5 (20 µM) completely recovered their tyrosinase activity, while those treated with CMC2.24 (20 µM) and CMC2.23 (20 µM) only partially recovered their tyrosinase activity, which could imply that longer recovery periods may be needed for cells to regain full tyrosinase activity after previous exposure to CMC2.23 or CMC2.24 at the higher concentration of 20 µM. However, longer recovery time points could not be tested using B16F10 cultures due to limitations caused by rapid cell growth and melanin production, at which point the cultures became over-confluent and detached after longer recovery periods. The higher concentrations of CMC2.23 (20 µM) and CMC2.24 (20 µM) may not contribute to delayed recovery, since the same concentration of the closely related inhibitor CMC2.5 (20 µM) could be removed with rapid complete recovery of the tyrosinase activity. This result might point to a structure-activity based effect on recovery of tyrosinase activity wherein the methoxycarbonyl group modification on β-diketone moiety of curcumin (as in CMC2.14 and CMC2.5) facilitated rapid recovery of the inhibited tyrosinase activity, while the phenylaminocarbonyl group modification (as in CMC2.24 and CMC2.23) contributed to delayed recovery of the inhibition of tyrosinase activity. Another explanation for differences in recovery after removal of drug could be that the compounds (CMC2.23 and CMC2.24) might have a higher intracellular uptake or might be sequestered in melanosomes via melanin-binding, which could result in their higher intracellular retention and a prolonged duration for the cells to expel out the compounds, resulting in delayed recovery; however, this hypothesis warrants further testing. We did not assess the cellular permeability or uptake of CMCs, but based on a published report, curcumin has been shown to be taken up and subsequently localized in the plasma membrane of cells [55], hence we expect that CMCs might also exhibit similar uptake profiles, especially since they have better solubility and stability than the parent compound curcumin.

Our data showed that CMCs displayed a spectrum of inhibition of melanogenesis, with CMC2.24 showing potent inhibition of α-glucosidase activity in a cell-free system that was superior to that achieved by PC. The data show that CMCs may inhibit melanogenesis through a novel mechanism in which the inhibition of tyrosinase glycosylation and maturation [45,56] is achieved by targeting α-glucosidase activity; this target has been previously reported for other anti-melanogenic compounds [57,58]. By inhibiting the enzyme, CMCs could affect tyrosinase glycosylation and maturation and hence reduce melanin synthesis. This is a novel mechanism for inhibiting melanogenesis and has not been reported yet for curcumin or its analogs.

MITF plays a key role in regulation of the survival and differentiation of melanocytes [59] and there have been reports of skin-lighteners that can reduce the levels of MITF or cause its degradation. MITF also has a pivotal role in regulation of melanosome transport by acting on the melanosomal proteins which form a complex in which myosin VA and Rab27A are joined by the effector melanophilin (MLPH) [60]. A study has documented that curcumin inhibited melanin production in human melanocytes by reducing protein levels of MITF after 48 h [61]. Our results of MITF protein levels in B16F10 cells treated with PC and CMCs showed a significant inhibition in protein levels by CMCs; this result suggests that CMCs might inhibit melanosome transport by downregulating MITF protein levels, although the evaluation of the effects of CMC on melanosome transport were not the focus of this study and warrant future investigation.

The ratio of dark-colored macromolecular pigment (eumelanin) and light-colored macromolecular pigment (pheomelanin) plays a role in skin pigmentation and a high intracellular sulfur concentration can divert the melanin synthesis pathway towards formation of pheomelanin. For example, thiols [62] and glutathione derivatives [63,64] cause skin-lightening by diverting the melanin synthesis pathway towards the formation of pheomelanin instead of eumelanin, resulting in a lower eumelanin/pheomelanin ratio. In the present study, we did not explore the effects of CMCs on the inhibition of melanin phenotype (eumelanin vs. pheomelanin). However, since curcumin has been reported to inhibit melanin synthesis without switching phenotype to pheomelanin in B16F10 cells [65], we speculate that CMCs might also possess the same attribute; however, this hypothesis requires quantitation of eumelanin and pheomelanin using spectrophotometric methods [64,66] or more sensitive method such as HPLC [67] and would be interesting for future investigation.

We have also conducted a preliminary evaluation of cytotoxicity (Figure S3A,B) and melanin inhibition in MNT-1 human melanoma cells (Figure S3C) for all the analogs including PC; our results showed that only CMC2.24 showed a significant attenuation of melanin levels at nontoxic concentration of 20 µM while the other three analogs and PC were ineffective. We did not further examine whether CMC2.24 might inhibit melanogenesis in other human tyrosinase-rich cell lines and normal human melanocytes, as that was beyond the scope of this study, and further studies are currently underway. As CMC2.24 has already demonstrated safety in vivo, using models of diabetes and periodontitis, as well as stability and bioavailability that were both superior to curcumin, our results of greatest inhibitory effects on tyrosinase activity and melanin production by CMC2.24 establish that this compound merits use for repurposing for the treatment of hyperpigmentation disorders. 

## 5. Conclusions

Our results provide a proof-of-principle for the novel use of the CMCs for inhibiting melanogenesis since at low micromolar concentration range (5–25 µM), the CMCs (CMC2.24, CMC2.23 and CMC2.5) were superior to parent compound PC, which was ineffective at diminishing melanogenesis at these low concentrations. The mechanisms of action of the CMCs include direct effects on tyrosinase enzyme activity, as seen in cell-free assays, as well as inhibitory effects on melanogenesis at the cellular level, effects on MITF protein levels and cAMP levels. Particularly, CMC2.24 could be repurposed as a drug for the treatment of hyperpigmentation disorders in dermatology or as an adjuvant to depigment melanomas for therapeutic purposes since toxicology studies have deemed it to have a high safety profile in other clinical applications. In addition, CMC2.5 and CMC2.23 hold promise as candidates for use in skin-lightening cosmetic formulations for dark-skinned individuals. Further studies on testing effects of CMCs on primary human melanocytes are currently ongoing.

## 6. Patents

S.G. and S.R.S. are listed as inventors on the patent application describing the use of curcumin analogs for inhibition of human melanogenesis (U.S. Patent 10,300,000). F.J. is listed as inventor on patent applications describing some of the compounds in this study. These patent applications have been fully assigned to their institutions, the Research Foundation of Stony Brook University and to Chem-Master International, Inc.

## Data Availability

Not applicable.

## Supplementary Materials

The following are available online at https://www.mdpi.com/article/10.3390/biom11050674/s1, Table S1: IC_50_ of compounds, PC and CMC2.24, compared with KA in mushroom tyrosinase assay. Table S2: Kinetic parameters of inhibition of mushroom tyrosinase by compounds PC, CMC2.24, and CMC2.23. Figure S1: Representative dose-response plots of IC_50_ determination of monophenolase and diphenolase inhibition for compounds PC, CMC2.24, and Kojic Acid. Figure S2: Melanin content levels under hormone-stimulated conditions in B16F10 cells treated with different concentrations of PC and CMCs showing (A) panel of cell pellets and; (B) intracellular melanin levels expressed as % of control in lysates. KA (500 μM) was used as positive control and control was treated with 0.16% DMSO. Data are mean ± SD of duplicate measurements. Figure S3: Viability of MNT-1 cells treated with (A) PC, CMC2.14 and CMC2.5 and (B) CMC2.23 and CMC2.24 for a duration of 48 h evaluated by MTS assay; Data for (A,B) are mean ± SD of 3–4 determinations; (C) Melanin content assay in cultures of MNT-1 cells treated for 48 h with different concentrations of PC and CMCs, Data are mean ± SD of values combined from two independent experiments. * *p* < 0.05 vs. control (Ctrl); One-way ANOVA with Tukey’s test.
