# Supplementary material for: Novel Chemically Modified Curcumin (CMC) Derivatives Inhibit Tyrosinase Activity and Melanin Synthesis in B16F10 Mouse Melanoma Cells
Source: Biomolecules. 2021 Apr 30;11(5):674. doi: 10.3390/biom11050674 (PMC8145596; doi:10.3390/biom11050674)
Supplement: Supplementary file 1 [file biomolecules-11-00674-s001.zip › biomolecules-1191708-supplementary.pdf]

SUPPLEMENTARY INFORMATION

Novel chemically-modified curcumin (CMC) derivatives inhibit tyrosinase activity and melanin synthesis in B16F10 mouse melanoma cells

Shilpi Goenka[1, *], Francis Johnson[2,3,4,] and Sanford R. Simon[1,5,6]

[1]Department of Biomedical Engineering,
[2]Department of Chemistry,
[3]Department of Pharmacological Sciences,
[4]Chem-Master International, Inc., Stony Brook, NY, USA
[5]Department of Biochemistry and Cellular Biology,
[6]Department of Pathology, Stony Brook University, Stony Brook, NY, USA

*** Correspondence**
Shilpi Goenka
Department of Biomedical Engineering
Stony Brook University, Stony Brook, NY 11794-5281.
Email: shilpi.goenka@stonybrook.edu

Table S1: IC_{50} of compounds, PC and CMC2.24, compared with KA in mushroom tyrosinase assay

Compound	IC_{50} for Monophenolase Inhibition (μM)	IC_{50} for Diphenolase inhibition (μM)
KA	23.93 ± 0.96	70.87 ± 6.10
PC	66.99 ± 2.40	298.97 ± 35.65
CMC2.24	25.05 ± 1.18	37.17 ± 3.70

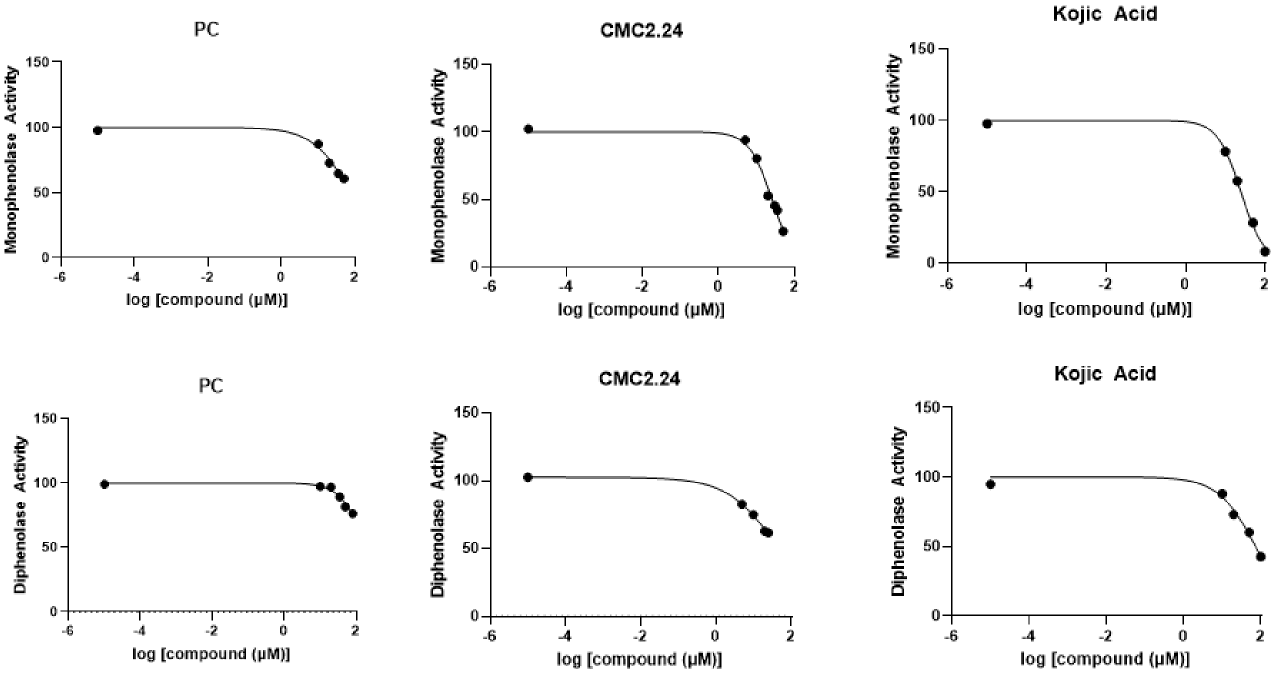

Figure S1: Representative dose-repose plots of IC_{50} determination of monophenolase and diphenolase inhibition for compounds PC, CMC2.24 and Kojic acid.

Table S2: Kinetic parameters of inhibition of mushroom tyrosinase by PC, CMC2.24, and CMC2.23

		Monophenolase (L-TYR)			Diphenolase (L-DOPA)		
**Compound**	**Inhibition Mechanism**	**K_i (µM)**	**K_m (mM)**	**V_{max} (mM/min)**	**K_i (µM)**	**K_m (mM)**	**V_{max} (mM/min)**
PC	Competitive	7.74	0.06	25.1	45.15	0.4	44.67
	Noncompetitive	50.76	0.11	29.97	163.7	0.5	48.28
	Uncompetitive	40.2	0.13	29.13	129	0.54	47.52
CMC2.24	Competitive	4.56	0.08	24.62	7.83	0.33	36.0
	Noncompetitive	16.47	0.21	32.20	42.11	0.61	43.69
	Uncompetitive	23.64	0.15	29.92	32.54	0.76	41.99
CMC2.23	Competitive	27.68	0.19	22.34	73.2	0.34	38.0
	Noncompetitive	79.3	0.25	24.96	410.4	0.40	39.05
	Uncompetitive	50.65	0.30	26.51	419.12	0.41	39.0

K_i- Inhibition constant; K_m- Michaelis constant; V_{max}- maximum velocity

I. *Melanin content measurement in αMSH-stimulated B16F10 cells.*

B16F10 cells were seeded at 1 x 10^5 cells/ well in in 12-well plates and incubated for 24 hours. The compounds were then added in presence or absence of αMSH (100 nM), and further incubated for another 48 hours. For evaluation of melanin content, cells were harvested, washed in PBS and then 250 μL of 1N NaOH was added and heated to 70 °C to solubilize melanin. The aliquots were then transferred to a 96-well plate and absorbance was read at 475 nm using microplate reader. The absorbance of melanin was normalized to the total protein contents and reported as % of control.

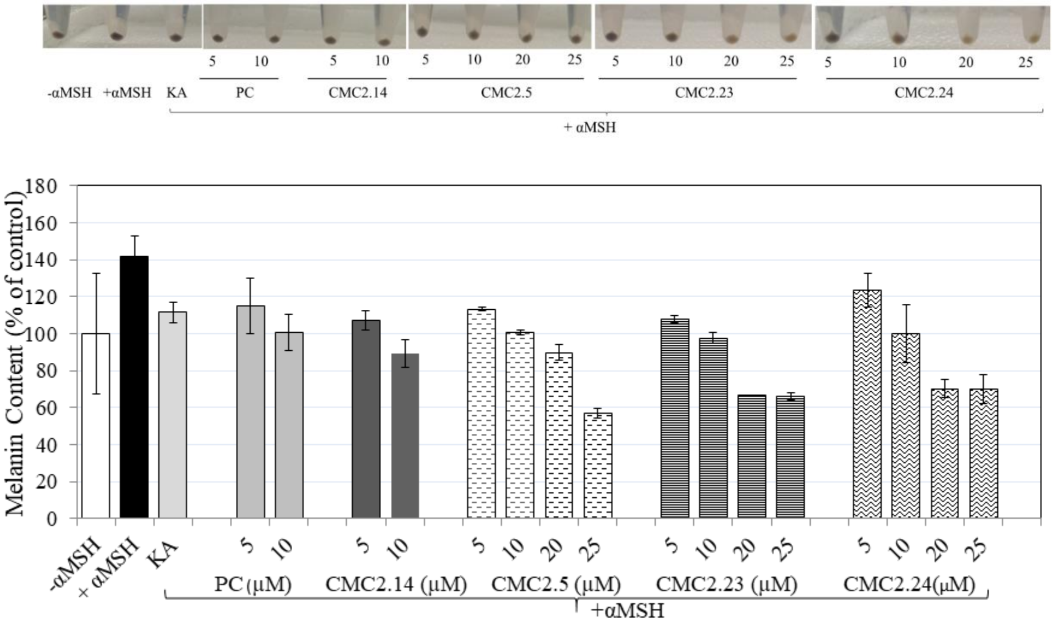

Figure S2: Melanin content levels under hormone-stimulated conditions in B16F10 cells treated with different concentrations of PC and CMCs showing A) panel of cell pellets and; B) intracellular melanin levels expressed as % of control in lysates. KA (500μM) was used as positive control and control was treated with 0.16 % DMSO. Data are mean ± SD of duplicate measurements.

II. *Experiments in MNT-1 human melanoma cells*

a. *Cell Culture*

MNT-1 cells (kindly provided by Dr. Michael Marks, University of Pennsylvania) were cultured using DMEM supplemented with 1 % MEM (minimum essential medium), 18 % HI-FBS, 10 % AIM-V medium (Gibco) and 1 % antibiotic mixture.

b. *Cytotoxicity assay*

MNT-1 human melanoma cells (2×10^4 cells/ well) were seeded in a 96-well plate and cultured for 96 h. After this, the test compounds were added in complete medium and cultures were maintained for 48 h, followed by conducting the MTS assay to detect cytotoxicity similar to methods reports in main text. The results were expressed as % normalized to untreated control group.

c. *Melanin content assay*

MNT-1 human melanoma cells were seeded at 2.2×10^5 cells/well in 12-well plates and incubated for 24 hours. The test compounds prepared in DMSO were added while the controls were treated with 0.1% DMSO, and the cultures were incubated for another 48 hours. After this step, the cells were processed for the estimation of melanin content similar to the method reported in main text methods section. Briefly, the absorbance of melanin was measured at 475 nm and was normalized to the total protein contents and reported as relative melanin levels as a % of control.

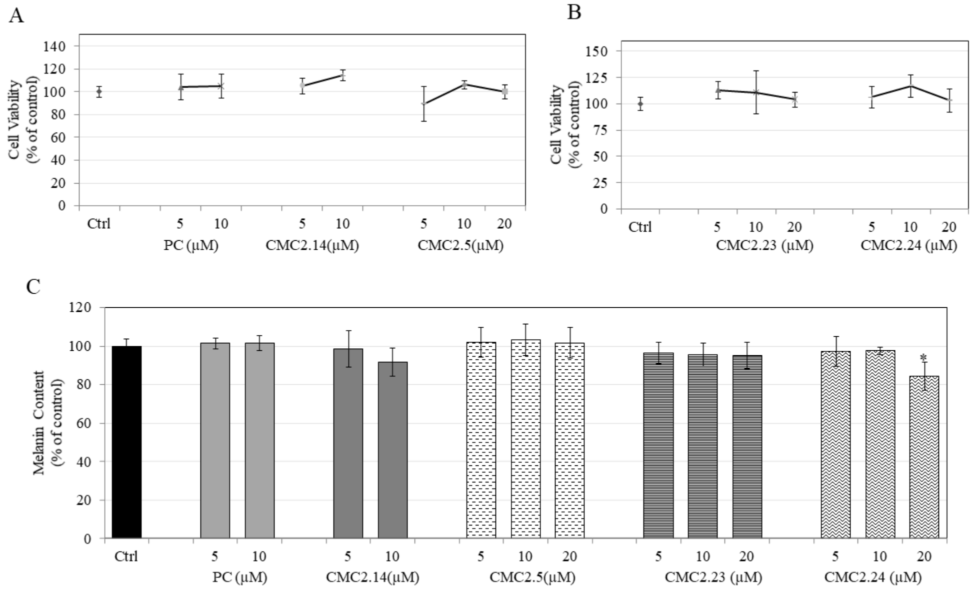

Figure S3: Viability of MNT-1 cells treated with **A)** PC, CMC2.14 and CMC2.5 and **B)** CMC2.23 and CMC2.24 for a duration of 48 h evaluated by MTS assay; Data for A)-B) are mean ± SD of 3-4 determinations; **C)** Melanin content in cultures of MNT-1 cells treated for 48 h with different concentrations of PC and CMCs, Data are mean ± SD of values combined from two independent experiments. * $p < 0.05$ vs. control (Ctrl); One-way ANOVA with Tukey's test.
